# Gestational diabetes and risk of perinatal depression in low- and middle-income countries: a meta-analysis

**DOI:** 10.3389/fpsyt.2024.1331415

**Published:** 2024-02-12

**Authors:** Yuqing Jin, Chengkai Wu, Wanlin Chen, Jingsong Li, Haiteng Jiang

**Affiliations:** ^1^ Affiliated Mental Health Center & Hangzhou Seventh People’s Hospital and School of Brain Science and Brain Medicine, Zhejiang University School of Medicine, Hangzhou, China; ^2^ Research Center for Healthcare Data Science, Zhejiang Laboratory, Hangzhou, China; ^3^ Department of Psychology and Behavioral Sciences, Zhejiang University, Hangzhou, Zhejiang, China; ^4^ Engineering Research Center of Electronic Medical Record (EMR) and Intelligent Expert System, Ministry of Education, College of Biomedical Engineering and Instrument Science, Zhejiang University, Hangzhou, China; ^5^ Liangzhu Laboratory, Ministry of Education (MOE) Frontier Science Center for Brain Science and Brain-machine Integration, State Key Laboratory of Brain-machine Intelligence, Zhejiang University, Hangzhou, China

**Keywords:** mental disorders, gestational diabetes, meta-analysis, pregnancy, perinatal depression, developing countries

## Abstract

**Background:**

The relationship between gestational diabetes (GDM) and the risk of depression has been thoroughly investigated in high-income countries on their financial basis, while it is largely unexplored in low- and middle- income countries. This meta-analysis aims to assess how GDM influences the risk of perinatal depression by searching multiple electronic databases for studies measuring the odds ratios between them in low- and middle-income countries.

**Methods:**

Two independent reviewers searched multiple electronic databases for studies that investigated GDM and perinatal mental disorders on August 31, 2023. Pooled odds ratios (ORs) and confidence intervals (CIs) were calculated using the random effect model. Subgroup analyses were further conducted based on the type of study design and country income level.

**Results:**

In total, 16 observational studies met the inclusion criteria. Only the number of studies on depression (n=10) satisfied the conditions to conduct a meta-analysis, showing the relationship between mental illness and GDM has been overlooked in low- and middle-income countries. Evidence shows an elevated risk of perinatal depression in women with GDM (pooled OR 1.92; 95% CI 1.24, 2.97; 10 studies). The increased risk of perinatal depression in patients with GDM was not significantly different between cross-sectional and prospective design. Country income level is a significant factor that adversely influences the risk of perinatal depression in GDM patients.

**Conclusion:**

Our findings suggested that women with GDM are vulnerable to perinatal depressive symptoms, and a deeper understanding of potential risk factors and mechanisms may help inform strategies aimed at prevention of exposure to these complications during pregnancy.

## 1 Introduction

Gestational diabetes (GDM) is defined as glucose intolerance with onset or first recognition during pregnancy and can affect up to 25% of women during pregnancy globally (1). As one of the most common pregnancy complications, GDM is related to both short- and long-term adverse health outcomes in women and their offspring. Women with GDM are more likely to have gestational hypertension, preeclampsia, emergency Caesarean delivery, and type 2 diabetes mellitus (2–4). Besides, increasing evidence also suggested the close relationship between GDM and the risk of mental disorders, with a predominant focus on the attention drawn to its association with depression (5–7). For instance, a recent meta-analysis in 10 cohort studies with a total population of 2,000,002 identified a significantly increased risk of developing postpartum depressive symptoms in women with GDM (8). The risk of depression in women with GDM is worth emphasizing, as physical health and mental health are tightly connected. When mental health problems coexist with physical health problems, health outcomes, disability, and costs tend to be much worse (9, 10).

However, the relationship between GDM and the risk of perinatal depression in low- and middle-income countries has only recently become the subject of interest. Accumulating evidence shows both the risks of physical and mental health vary based on income levels (11, 12). Moreover, high-income countries tend to have more healthcare budgets and distribute greater proportions of budgets on mental health treatment than low- and middle-income countries. Therefore, previous findings based on high-income countries were insufficient to guide disease treatment in low- and middle- income countries.

Recent research found a mental health-based “poverty trap”: poverty results in poor physical health and early-life conditions, which in turn leads to depression and anxiety disorders that could adversely affect individuals’ childhood development, productivity, women’s empowerment, as well as economic decision-making, and eventually reinforces poverty (9). Hence, understanding the link between physical and mental health, as well as how they interact with income, is an important next step for low- and middle-income countries. It not only allows countries to optimize the distribution of their healthcare budgets, but also reinforces them to escape the poverty trap and enhance economic gains. Therefore, the primary aim of this meta-analysis is to systematically investigate the association between GDM and the risk of perinatal depression in low- and middle-income countries; by doing this, we want to emphasize the importance of caring for depression among the GDM population, especially in low- and middle-income countries.

## 2 Material and methods

### 2.1 Literature search

Two investigators independently (YJ and CW) searched databases of Medline, EMBASE, Pubmed, Web of Science, and PsycINFO from inception until August 31, 2023. Search terms such as “gestational diabetes mellitus” and “mental disorders” were adapted from previous systematic reviews in the area (13–15). The complete list of the search terms used is presented in the **Supplementary File**. Forward and backward citation was also undertaken.

### 2.2 Study selection

Inclusion criteria were confined to peer-reviewed studies published in English or with sufficiently detailed English abstracts to extract relevant information, measuring both GDM and perinatal mental disorders. Perinatal mental disorders included depression, anxiety, psychotic or eating disorders diagnosed at antenatal (between conception and delivery) or postpartum (up to 1 year following delivery) period, as there were plausible mechanisms for an association between these disorders and GDM. The study type is either cohort (prospective or retrospective) or cross-sectional.

Exclusion criteria included studies conducted in countries classified as high-income by the World Bank. Additionally, studies from high-income regions of Hong Kong, Taiwan, and Macau were excluded from the analysis due to their distinct economic and healthcare conditions compared to mainland China. Furthermore, studies in which mental disorders were diagnosed prior to the onset of GDM were excluded. Finally, studies that did not report unadjusted odds ratios for the relationship between GDM and mental disorders, or did not provide sufficient data for the calculation of odds ratios, were excluded from the meta-analysis.

Following de-duplication, titles and abstracts were screened, followed by full-text screening by two independent reviewers. In total, 16 studies met the study’s inclusion criteria.

### 2.3 Data extraction

Data extraction was conducted by two independent reviewers (YJ and CW) and the following data were extracted: the last name of the first author, year of publication, country, sample size, study design, diagnostic criteria of exposure and outcome, the timing of outcome assessment (antepartum vs. postpartum), significant risk factors (BMI, age, occupation, etc.), and unadjusted odds ratios with corresponding 95% confidence intervals (CIs).

### 2.4 Risk of bias assessment

The quality of the selection, comparability, and outcome of the included studies was assessed using a pre-piloted modified Newcastle-Ottawa scale (16) (**Supplementary Table S1**). Two independent reviewers (YJ and CW) performed the quality assessment and scored the included studies. Scores for selection bias and measurement bias were of particular interest as most of the studies were of observational design. A study with a score of zero in any of the evaluation domains was categorized as high risk of bias. Otherwise studies were categorized as low to moderate risk (17, 18). A lower risk of bias indicates higher quality.

### 2.5 Data synthesis

Unadjusted ORs with 95% CIs were used as measures of the association as studies were adjusted for different covariates. If ORs for at least three studies were available for one mental disorder, a meta-analysis was performed (19). DerSimonian-Laird random effects model (20) was the most commonly used method in meta-analysis because it is especially useful for providing an overall effect estimate and characterizing the heterogeneity of effects across a series of studies. When the proportion of total variation in study estimates that is due to heterogeneity (denoting as *l* ^2^), it was decided a-priori such as 90% would preclude meta-analysis as this represents substantial heterogeneity (21). To evaluate the influence of each study, we conduct a sensitivity analysis by omitting each study individually and recalculating the pooled unadjusted ORs for the rest of the studies. All analyses were performed using STATA version 17 (22).

Subgroup analysis was performed for factors that could potentially impact the relationship between GDM and the risk of perinatal mental disorders. Potential factors include study type (prospective or cross-sectional studies), country income level, the timing of diagnostic (symptoms measured in antepartum or postpartum period) and mental disorder type. If ORs for at least three studies were available for each subgroup, a subgroup meta-analysis was additionally performed.

## 3 Results

### 3.1 Study characteristics

**Figure 1:**
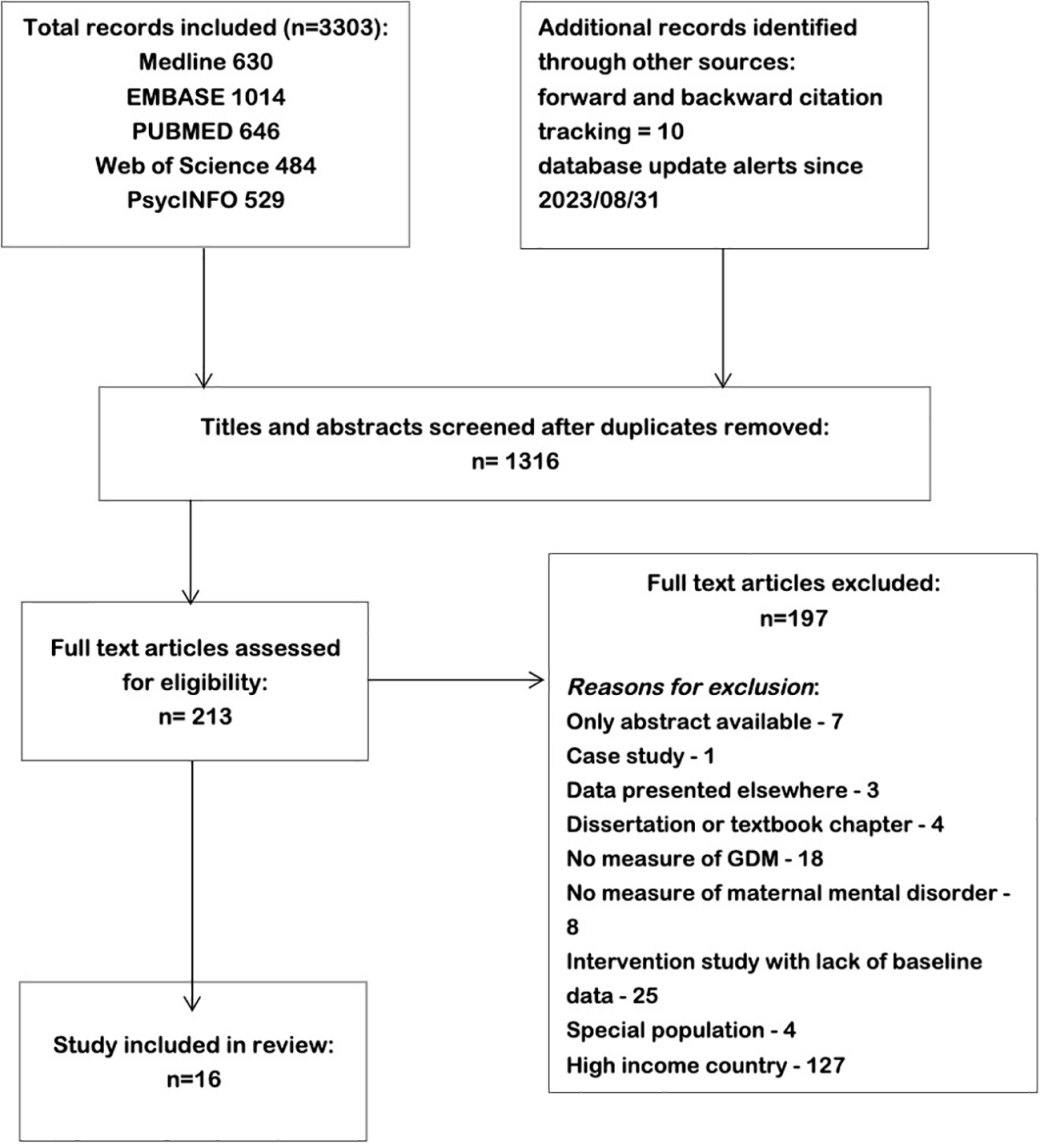
Study selection process for meta-analysis of studies on Gestational diabetes and risk of perinatal mental disorders in low- and middle-income countries.

As shown in **Figure 1**, we identified 1316 studies from five different electronic databases. During the initial screening by title and abstract, the majority of the articles were excluded for being conducted in high-income countries or intervention studies without baseline data.

Among the 16 studies included, 10 studies were eligible for meta-analysis, and 6 studies were only used for prevalence and risk factors analysis due to lack of unadjusted ORs. The characteristics of the included studies were summarized in **Table 1**.

**Table 1:** Characteristics of included studies.

Disorder	Depression	Anxiety
Total N (2 studies measures both depression and anxiety)	14	4
Study Design		
Cross-sectional	6	2
Prospective cohort	8	2
Income Category		
Low-income	1	0
Lower-middle-income	2	2
Upper-middle-income	11	2
Country		
Bangladesh	1	0
Brazil	1	0
China	6	1
India	1	1
Iraq	1	1
Peru	1	0
Sri Lanka	0	1
Turkey	2	0
Ethiopia	1	0

The most prevalent study design was prospective cohort (N=10) and 8 studies were cross-sectional. All of the studies were performed in low- or middle-income countries and 7 studies were from China. Diagnostic criteria for GDM include the International Classification of Diseases (ICD), oral glucose tolerance test, medical records, and self-report. Assessments for depressive symptoms or depression were based on the Edinburgh Postnatal Depression Scale (EDPS), the ICD, the Montogomery and Asberg Depression Rating Scale (MADRS), the Zung Self-Rating Depression Scale (SDS), The Beck Depression Inventory (BDI), and self-report. Assessment for anxiety symptoms or anxiety were based on the Self-Rating Anxiety Scale (SAS), the ICD, and MINI structured interview.

It is worth mentioning that in our original planned analysis, we intended to examine the relationship between GDM and a comprehensive range of perinatal mental disorders, encompassing depression, anxiety, psychotic, and eating disorders. However, the search results suggest current studies from low- and middle-income countries can only be found sufficient when they pertain to either depression or anxiety. Furthermore, among these perinatal mental disorders, only the quantity of literature on depression fulfilled the criteria for meta-analysis. Therefore, this study will primarily concentrate on examining the risk associated with perinatal depression in patients with GDM.

### 3.2 Risk of depression in patients with GDM

Out of 16 included studies, 10 studies measured diagnoses or symptoms of depression and were eligible for meta-analysis (23–32). Their respective characteristics and relevant findings were presented in **Table 2**.

**Table 2:** Summary of data provided by each study.

Author and year	Country	Study design and sample size	GDM measure	Mental disorder measure	Risk factors	Quality	Unadjusted OR(95%CI)	Type
Atlaw et al., 2022 (24)	Ethiopia	Prospective cohort, N=432 women GDM- 68	fasting capillary blood glucose between 92 and 125 mg/dL	The Edinburgh Postnatal Depression Scale	GDM: 1,3,5	Low to moderate risk of bias	^a^5.9 (3.04, 11.48)	Antenatal depression
Boggaram et al., 2017 (33)	India	Cross-sectional, N=100 women GDM- 11	Not specified	MINI structured interview during pregnancy (unknown if MINI ICD10 or DSM-IV) for anxiety disorders		High risk of bias	^c^3.33 (0.75,14.87)	Antenatal anxiety
Hassan et al., 2017 (23)	Iraq	Prospective cohort, N=100 GDM- 50	OGTT	BDI ≥ 20 at 24-36 weeks gestation		High risk of bias	Depression ^a^4.45 (1.68,11.81) Anxiety ^a^1.64 (0.74,3.66)	Antenatal depression or anxiety
Isik and Cetisli., 2022 (25)	Turkey	Cross-sectional study, N=237 women GDM- 104	Based on medical records	EPDS ≥12	GDM: 1,4,7,9	Low to moderate risk	Antenatal ^b^1.46 (0.85, 2.50) Postpartum ^b^1.35 (0.68, 2.66)	Antenatal and Postpartum depression
Keskin et al., 2015 (27)	Turkey	Prospective cohort, N=89 women GDM- 44	OGTT	Antepartum BDI (unknown what version) ≥17 after GDM diagnosis	GDM: 2	High risk of bias	^a^1.19 (0.41,3.43)	Antenatal depression
Larrabure-Torrealva et al., 2018 (29)	Peru	Cross-sectional study, N=1300 women GDM- 205	OGTT	Patient Health Questionnaire-9	GDM: 1,2,5	High risk of bias	^a^1.52 (1.09–2.12)	Antenatal depression
Li et al., 2022 (31)	China	Retrospective cohort, N=1043 women GDM - 313	OGTT	Edinburgh Postnatal Depression Scale (EPDS) ≥ 9	GDM: 1,2,3,5,7,8	Low to moderate risk of bias	1st trimester: ^a^0.65(0.44–0.94) 2nd trimester: ^a^0.86(0.49–1.53)	Antenatal depression
Natasha et al., 2015 (28)	Bangladesh	Prospective cohort, N=748 women GDM - 382	Plasma Glucose found ≥7.0 (WHO) or ≥5.3 mmol/L at Fasting, and ≥8.6 mmol/L at 2 h after 75 gm Glucose intake (ACOG), (which ever detected first)	Montogomery and Asberg Depression Rating Scale (MADRS)	GDM: 1,4,6	Low to moderate risk of bias	^a^3.02 (2.01, 4.53)	Antenatal depression
Singh et al.,2023 (32)	India	Prospective cohort, N=347 women GDM- 48	Seventy-five grams of glucose was given in 300 ml of water irrespective of fasting stage and blood glucose was measured by glucometer using reagent strips after two hours. The blood glucose level of ≥140 mg/dl after two hours of glucose load was taken as cut off for diagnosis of GDM.	EPDS ≥12	Depression: 1, 3, 4	Low to moderate risk of bias	^b^1.71(0.70,4.19)	Postpartum depression
Song et al, 2004 (26)	China	Prospective cohort, N=104 women GDM- 50	OGTT	SDS (Zung Self-rating depression scale) during pregnancy ≥41		High risk of bias	^a^3.53 (1.04,11.93)	Antenatal depression
Tang, Yi et al, 2020 (30)	China	Prospective cohort, N=1426 women GDM- 533	OGTT	self-rating anxiety scale,SAS≥50 as anxiety and self-rating depression scale,SDS ≥53 as depression		Low to moderate risk of bias	Anxiety: ^b^1.22 (0.82, 1.81) Depression: ^b^0.83(0.58,1.20)	Antenatal anxiety and depression

GDM, gestational diabetes; OR, odds ratio; OGTT, oral glucose tolerance test.

Risk factors: 1, age; 2, BMI; 3, occupation; 4, educational level; 5, family history of diabetes; 6, history of hypertension; 7, parity; 8, gravidity; 9, social support.

a Estimate given in paper.

b Derived from data in paper.

c Data provided by study author.

**Figure 2:**
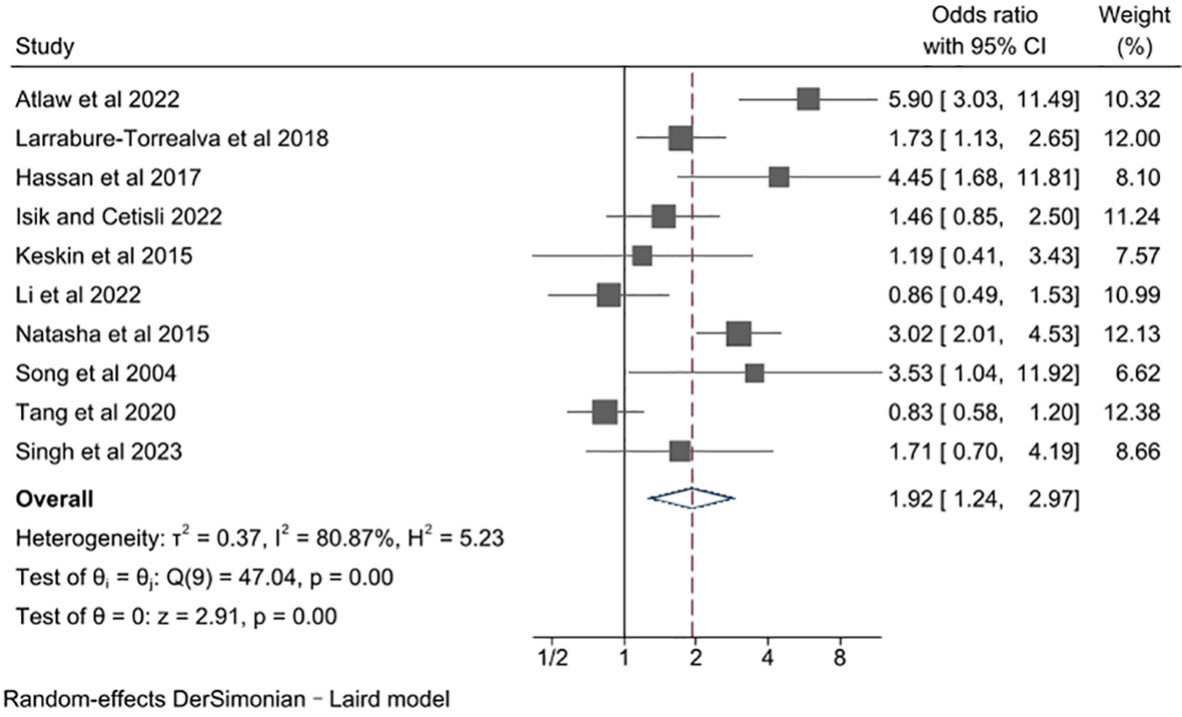
Meta-analysis of studies examining the association between gestational diabetes and risk of perinatal depression.

The unadjusted ORs varied from 0.83 to 5.90 across studies (**Figure 2**). Among the 10 studies, 8 studies found a significant increase in risk of depression, while 2 studies reported no association. Pooling together, women with GDM compared with the control group had a notably increased risk of developing perinatal depressive symptoms (pooled unadjusted OR= 1.92, 95% CI 1.24, 2.97). There was a high degree of heterogeneity across studies (*l* ^2^ = 80.87%, P for heterogeneity = 0.00).

The following 6 studies were not included in the meta-analysis for having no unadjusted ORs available as effect estimates. Dame et al. reported the proportion of women with antenatal depression among GDM women (proportion = 31%) (34). Mak et al. found that the 3 months postpartum EPDS score was significantly higher in women with GDM than those without GDM (EPDS in GDM group=2.1, EPDS in control group=1.5, p-value <0.001) (35). Chen et al. and Peng et al. provided GDM prevalence and treated depression as exposure (36, 37). Dai et al. aggregated depression, anxiety and obsessive-compulsive disorders into one measure and reports the prevalence of GDM in psychiatric and healthy control group (prevalence in psychiatric group = 20.7%, prevalence in healthy control group=6.1%) (38). Lastly, Levy-Shiff et al. found no association between GDM and depressive symptoms in second trimester (BDI score in GDM group=6.70, BDI score in control group=6.59, p-value=0.42) (39).

### 3.3 Study type influence in risk of depression in patients with GDM

**Figure 3:**
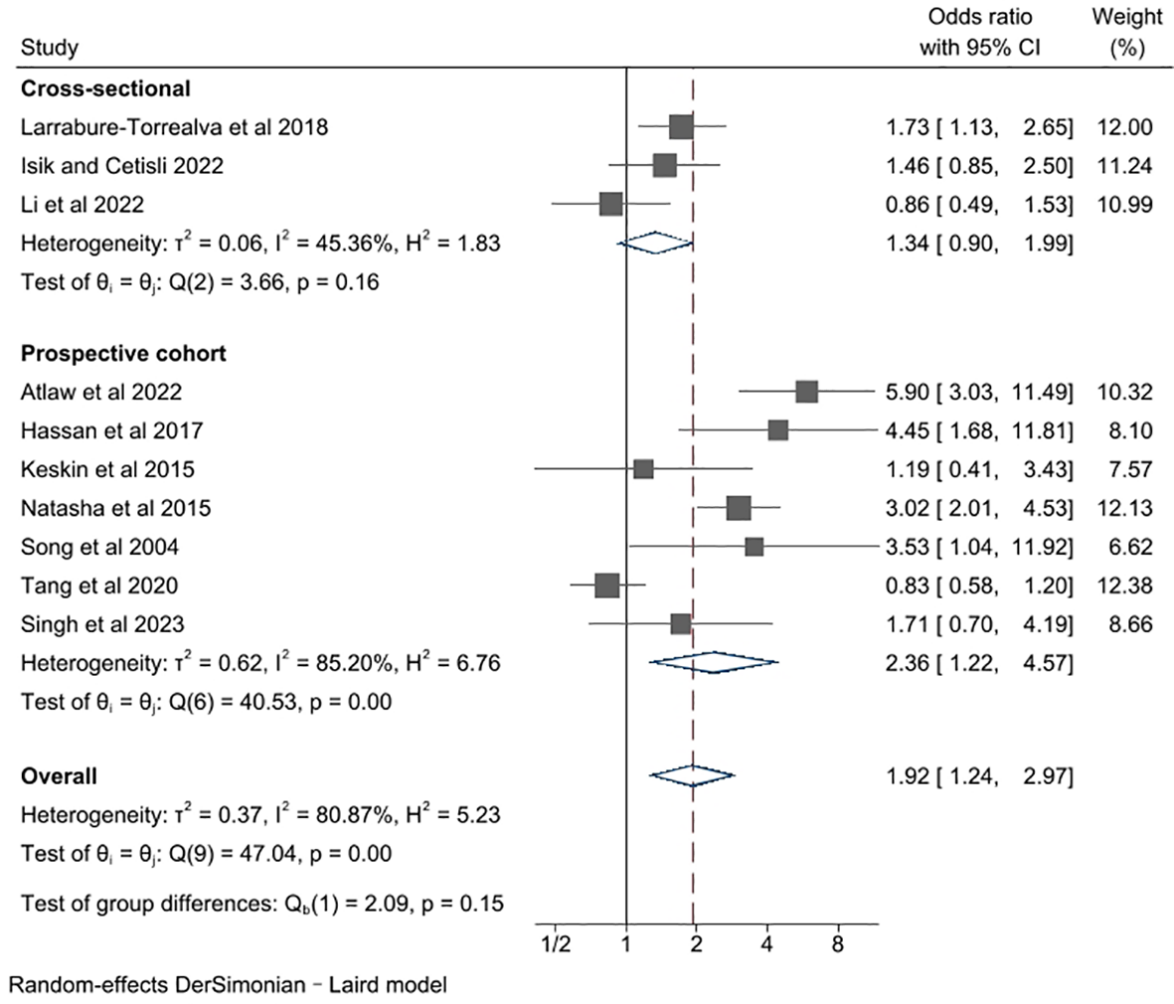
Subgroup meta-analysis of studies examining the association between gestational diabetes and risk of perinatal depression according to study design type.

In this section, we investigated the impact of study type on the reported results of the relationship between GDM and the risk of perinatal depression, as a prior study observed significant variations in associations across different study types (8), by performing a subgroup analysis. In the subgroup analysis, only the difference between cross-sectional and prospective studies was analyzed (**Figure 3**), as there were not enough retrospective studies presented. The pooled unadjusted ORs for cross-sectional and prospective study design were 1.34 (95% CI 0.90,1.99) and 2.36 (95% CI 1.22, 4.57) respectively. Cross-sectional studies had lower estimates than prospective studies, but the difference in pooled unadjusted ORs across different study design was not substantial (P for group difference = 0.15). There was no evidence of heterogeneity in cross-sectional cohort studies (*l* ^2^ = 45.36%, P for heterogeneity = 0.16), and a high degree of heterogeneity in prospective cohort studies (*l* ^2^ = 85.20%, P for heterogeneity = 0.00). Sensitivity analysis did not identify studies that had substantial influences on the overall effect estimate, with pooled unadjusted ORs ranging from 1.66 to 2.14.

### 3.4 Income influences in risk of depression in patients with GDM

**Figure 4:**
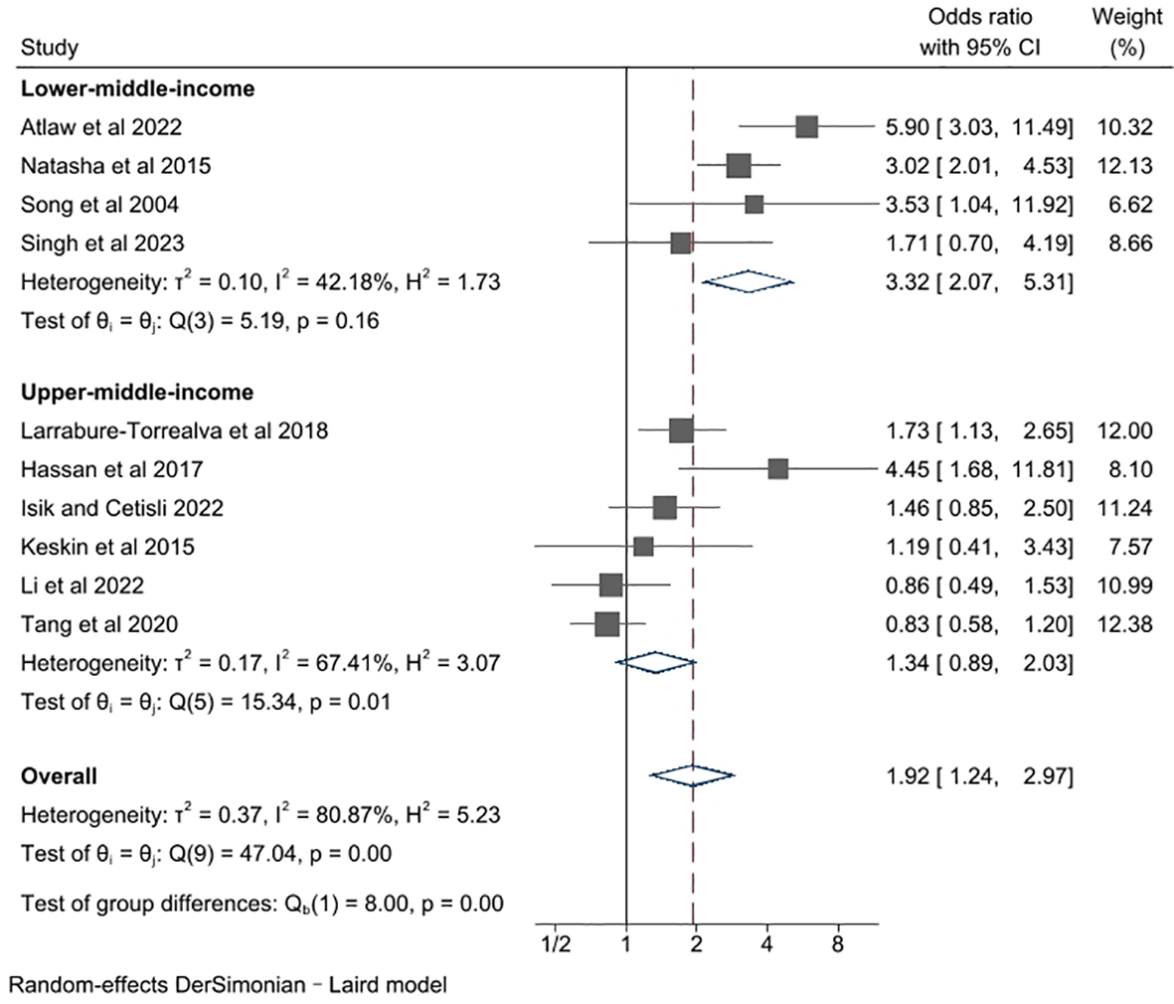
Subgroup meta-analysis of studies examining the association between gestational diabetes and risk of perinatal depression according to country income level.

In this section, we proceeded to conduct a subgroup analysis based on income levels (**Figure 4**). Specifically, the studies were divided into subgroups of lower-middle-income and upper-middle-income, according to the World Bank’s yearly classification of national income level. That means the studies conducted in a same country, mainly China in our analysis, would be grouped differently due to the income level at their publication year. As a major result, the association between GDM and depression was found to be remarkably influenced by income levels of studied countries (P for group difference = 0.00). The pooled unadjusted ORs for studies performed in lower-middle- and upper-middle-income countries were 3.32 (95% CI 2.07, 5.31) and 1.34 (95% CI 0.89. 2.03), respectively. There was no evidence of heterogeneity in studies from lower-middle-income countries (*l* ^2^ = 42.18%, P for heterogeneity = 0.16), and a notable degree of heterogeneity in studies from upper-middle-income countries (*l* ^2^= 67.41%, P for heterogeneity = 0.01). Besides, we found that the risk of depression in women with GDM is significantly higher in lower-middle-income countries compared to that in upper-middle-income countries, suggesting country income level is a significant factor that adversely influences the risk of perinatal depression in middle-income countries. It is unfortunate that data from low-income countries were insufficient to take part in this subgroup analysis, which could have made the analysis result more comprehensive.

## 4 Discussion

### 4.1 Main findings

Our meta-analysis differed from previous literature with an emphasis on studies conducted in low- and middle-income countries. Pooled unadjusted ORs for risk of perinatal depression was 1.92 (95% CI 1.24, 2.97), indicating that women with GDM have elevated risk of depression than those without GDM. This finding was in accordance with past researches in high income countries (8, 40). Furthermore, the pooled unadjusted ORs was substantially higher in studies conducted in lower-middle-countries than that in upper-middle-income countries, which supports our hypothesis that poverty exposes women to adverse mental and physical conditions. Among the included studies, one study (23) in Iraq and another study in Ethiopia (24) have notably higher unadjusted ORs (OR=4.45, 95% CI 1.68, 11.81 and OR=5.90, 95% CI 3.03, 11.49) compared to other studies in the same country income category. We speculated the elevated risk of depression was linked to constant armed conflicts in the regions. Moreover, it should be pointed out that the number of studies in anxiety disorder and other mental illness did not meet our standard to conduct meta-analysis, leaving opportunities for future research in low- and middle-income countries.

### 4.2 Potential mechanisms

The mechanism underlying the relationship between GDM and the risk of perinatal depression is unclear. Previous literature on type 2 diabetes speculated that perinatal depression resulted from biochemical changes directly due to GDM or from the psychological factors related to GDM or its treatment (41). There is also evidence suggesting that diabetes and depression may share common biological risk factors. For example, dysregulation of the hypothalamic-pituitary-adrenal (HPA) axis has been observed in people with either diabetes or depression (42, 43). Women with GDM are more prone to experience increased inflammation and adipokine concentration, which are also related to depression as well (44, 45). The event of having GDM itself could also result in depressive mood. In addition, we found that GDM and mental disorders shared several common risk factors, including age, education level, and occupation. Women with elder maternal age or unemployed women and housewives are more likely to have GDM and mental disorders (**Table 2**). Besides, a number of studies found that depressive symptoms were related to difficulties in adaption to diabetic complications and adverse obstetric outcome, including caesarean delivery and preterm delivery (46, 47). Moreover, insufficient nutritional support is also speculated to be associated with mental illness and GDM (48, 49). Studies have indicated a consistent correlation between lower income levels and inferior diet quality. (50, 51). Compared to individuals with higher income, those with lower income consume fewer fruits and vegetables, a greater amount of sugar-sweetened beverages, and have a lower overall diet quality (52, 53). Based on the theory of social causation, the condition of poverty could cause depression through financial stress, decreased social capital and inferior diet (54).

### 4.3 Strengths and limitations

To our knowledge, this is the first study that has thoroughly reviewed the literature in low- and middle-income countries and meta-analyzed the risk of perinatal depression in women with GDM. Since effect estimate and symptoms of depression may vary across subgroups, our meta-analysis was also grouped by study design and country income level.

Most of the included studies only provided unadjusted ORs, which may inflate the estimates for risk of depression. A few studies indicated that BMI and ethnicity may moderate the impact of perinatal depression, but information related to these confounders were often missing from studies (55, 56). Furthermore, previous literature found that obesity, level of glycemic control and GDM management strategies (insulin vs. diet intervention) may also have an impact on depression (57–59). Despite acknowledging the potential moderating effect of these variables on perinatal depression, the lack of detailed reporting hindered our ability to conduct a robust subgroup analysis.

Nearly half of the studies were identified as high risk of bias. Studies at high risk of bias mostly lack information regarding sample selection process or GDM diagnostic criteria. There was a high degree of heterogeneity among included studies. The source of heterogeneity came from both depression and GDM. Moreover, the screening tools of perinatal depression and GDM varied across studies. For depression evaluation, there were multiple assessment tools including EPDS, BDI, and Patient Health Questionnaire-9, and there is a lack of consensus on the optimal cut-off point in the literature. For instance, the cut-offs for EPDS were 9, 10, and 12 in three included studies. The screening time of postpartum depression include 1-month, 3-months, and 6-months postpartum. Previous studies also have contradictory results regarding 6-months depressive scores (35, 60). For GDM diagnosis, two studies used self-reported data, which may add to the risk of information bias.

### 4.4 Implications

A future potential and urgent area for research is the investigation of relationships between GDM and the risk of mental disorders other than depression in low- and middle-income countries. Current studies in less common mental disorders, such as eating disorders and bipolar disorder, were mostly performed in high-income countries. Current studies independently found that the prevalence of GDM and mental disorders was both higher in resource-constrained countries (61, 62), but the relationship between them are still relatively unexplored. Research in resource-constrained countries is speculated to have an important impact, as we found in this study on depression that the severity of mental disorders could be significantly negatively correlated to country income level. The research would also be important from both social and healthcare contexts because mental health problems can cause adverse consequences for women, their infants, and even the larger families. Addressing barriers in nutrition education and counselling, diet intervention, antenatal and postpartum care services, as well as emotional support services may contribute to improve health outcomes of pregnant women in low- and middle-income countries. During future investigations, we also emphasize a greater understanding of the underlying mechanism between GDM and depression, for it is essential for interventions to reduce not only the risk of depression but also other complications.

## 5 Conclusion

In this study, we performed a meta-analysis to examine the risk of perinatal depression among individuals diagnosed with GDM in low- and middle-income countries. We searched for studies on various mental disorders, but only identified sufficient research on depression that met the criteria for inclusion in our meta-analysis. This finding underscores the limited amount of research available on perinatal mental disorders in low- and middle-income countries and emphasizes the urgent need for further studies in this area.

Focusing specifically on perinatal depression, we found a significant increase in the likelihood of experiencing depressive symptoms in individuals with GDM. This finding emphasizes the importance of managing GDM, as doing so can help reduce adverse obstetric outcomes. Additionally, we found that the risk of depression in women with GDM is significantly higher in lower-middle-income countries compared to that in upper-middle-income countries, indicating country income level is a significant factor that adversely impacts the risk of depression in middle-income countries. The implications of this study are particularly relevant for low- and middle-income countries, as depression can directly impact individuals’ economic decision-making and productivity, potentially leading to increased poverty. Therefore, addressing perinatal mental health issues, especially in the context of GDM, is crucial for improving overall well-being and socio-economic outcomes. A deeper understanding of the relation and mechanisms between GDM and depression may help to identify the risk of depression at an early stage and reduce obstetric complications.

## Data availability statement

The original contributions presented in the study are included in the article/**Supplementary Material**. Further inquiries can be directed to the corresponding authors.

## Author contributions

YJ: Writing – original draft, Writing – review & editing, Data curation, Formal analysis. CW: Data curation, Writing – review & editing. WC: Writing – review & editing. JL: Writing – review & editing. HJ: Funding acquisition, Writing – review & editing.
