# Supplementary material for: Gestational diabetes and risk of perinatal depression in low- and middle-income countries: a meta-analysis
Source: Front Psychiatry. 2024 Feb 12;15:1331415. doi: 10.3389/fpsyt.2024.1331415 (PMC10897974; doi:10.3389/fpsyt.2024.1331415)
Supplement: Supplementary file 1 [file DataSheet_1.docx]

Supplementary material 1. Search terms

1. MeSH terms- diabetes mellitus, diabetes, gestational (in Medline), gestational diabetes (in PsycINFO).
2. gestation* adj2 diabet*
3. GDM
4. 1 OR 2 OR 3
5. Exploded MeSH terms- mental disorders (in Medline and PsycINFO) and mental disease (in EMBASE)
6. MeSH terms- mental health (in EMBASE, Medline and PsycINFO)
7. psych* adj2 (problem* OR disorder* OR ill* OR health)
8. mental adj2 (problem* OR disorder* OR ill* OR health)
9. anxiety adj2 (problem* OR disorder*)
10. stress disorder
11. panic
12. obsessi*
13. compulsi*
14. OCD
15. obsessive compulsive disorder
16. PTSD
17. somat* adj disorder
18. adjustment disorder
19. dissociat* adj disorder
20. schizo*
21. psychosis
22. psychotic
23. delusion* adj disorder
24. mood adj2 (problem* OR disorder*)
25. affective adj2 (problem* OR disorder*)
26. BPAD
27. bipolar
28. mania
29. manic
30. cyclothymi*
31. depression
32. depressive
33. dysthymi*
34. Exploded MeSH terms- eating disorder(s) (in EMBASE and PsycINFO), feeding and eating disorders (in Medline)
35. binge adj eat*
36. anorex*
37. bulimi*
38. eat* adj2 disor*
39. compulsive adj2 (eat* OR vomit* OR purg*)
40. dysthymic disorder
41. behaviour control
42. psychological phenomena
43. maternal health
44. 5 OR 6 OR 7 OR 8 OR 9 OR 10 OR 11 OR 12 OR 13 OR 14 OR 15 OR 16 OR 17 OR 18 OR 19 OR 20 OR 21 OR 22 OR 23 OR 24 OR 25 OR 26 OR 27 OR 28 OR 29 OR 30 OR 31 OR 32 OR 33 OR 34 OR 35 OR 36 OR 37 OR 38 OR 39 OR 40 OR 41 OR 42 OR 43
45. 4 AND 44
