# Supplementary material for: Gestational diabetes and risk of perinatal depression in low- and middle-income countries: a meta-analysis
Source: Front Psychiatry. 2024 Feb 12;15:1331415. doi: 10.3389/fpsyt.2024.1331415 (PMC10897974; doi:10.3389/fpsyt.2024.1331415)
Supplement: Supplementary file 2 [file Table_1.docx]

| Domain | Score (0- high risk of bias, 1- medium risk of bias, 2- low risk of bias) | Comments |
| --- | --- | --- |
| **STUDY DESIGN Total= /2** | | |
| **Suitability of study design for review question**   1. unable to ascertain design of study 2. case control, retrospective cohort or cross-sectional study 3. prospective cohort, interventional or population-level study |  |  |
| **SELECTION BIAS Total= /4** | | |
| **Representativeness of sample**  How were participants recruited? What was the sampling frame?  Could there be any bias from inclusion or exclusion criteria?   1. selection criteria exclude participants of possible interest to the study question or information about the sampling process is not provided 2. some exclusion criteria which could limit the population being studied 3. general population/broad sampling frame |  |  |
| **Participation rates**   1. less than 60% agreed to participate or not reported 2. 60-79% agreement 3. 80-100% agreement or N/A for population-based data |  |  |
| **ATTRITION BIAS Total= /2** | | |
| **Loss to follow up/drop out during the study**  What proportion of those originally recruited completed the study?   1. less than 60% or not reported 2. 60-79% completed 3. 80-100% completed or the study was of a design as to make this N/A (e.g. retrospective cohort) |  |  |
| **MEASUREMENT BIAS Total= /4** | | |
| **Measure of gestational diabetes**  Was this by self-report or clinical diagnosis?   1. no description given or for population-level data, no diagnostic criteria/data extraction algorithm is provided 2. self-report or criteria for diagnosis not specified 3. clinical diagnosis and diagnostic criteria are specified |  |  |
| **Measure of mental disorder**  Was this by clinical interview or screening tool? Is the screening tool validated?   1. no description given, screening tool is not validated or for population-level data or no diagnostic criteria/data extraction algorithm is provided 2. well validated screening tool 3. diagnostic measure |  |  |
| **Comparability Total= /2** | | |
| **Confounders**   1. No attempt to correct, control or adjust for confounding factors 2. Only one important confounder was controlled for in the deign or analysis 3. More than one confounder controlled or adjusted for |  |  |
